# Supplementary material for: Modeling glioblastoma heterogeneity as a dynamic network of cell states
Source: Mol Syst Biol. 2021 Sep 16;17(9):e10105. doi: 10.15252/msb.202010105 (PMC8444284; doi:10.15252/msb.202010105)
Supplement: Supplementary file 6 — Source Data for Figure 5 [file MSB-17-e10105-s004.zip › Figure5A_sourcedata/GSEA_3071/hallmarks_stateII.GseaPreranked.1623742536692/HALLMARK_XENOBIOTIC_METABOLISM.html]

Details for gene set HALLMARK\_XENOBIOTIC\_METABOLISM[GSEA]

|  || Dataset | state23071 |
| Phenotype | NoPhenotypeAvailable |
| Upregulated in class | na\_neg |
| GeneSet | HALLMARK\_XENOBIOTIC\_METABOLISM |
| Enrichment Score (ES) | -0.22882846 |
| Normalized Enrichment Score (NES) | -1.0514728 |
| Nominal p-value | 0.39856374 |
| FDR q-value | 0.7437337 |
| FWER p-Value | 1.0 |
Table: GSEA Results Summary

  

Fig 1: Enrichment plot: HALLMARK\_XENOBIOTIC\_METABOLISM      
 Profile of the Running ES Score & Positions of GeneSet Members on the Rank Ordered List

  

| PROBE | GENE SYMBOL | GENE\_TITLE | RANK IN GENE LIST | RANK METRIC SCORE | RUNNING ES | CORE ENRICHMENT || 1 | MTHFD1 |  |  | 173 | 0.914 | -0.0067 | No |
| 2 | SERPINE1 |  |  | 299 | 0.756 | -0.0078 | No |
| 3 | HPRT1 |  |  | 351 | 0.709 | 0.0076 | No |
| 4 | MT2A |  |  | 393 | 0.682 | 0.0244 | No |
| 5 | IGFBP4 |  |  | 401 | 0.678 | 0.0494 | No |
| 6 | TMEM97 |  |  | 403 | 0.676 | 0.0758 | No |
| 7 | PDLIM5 |  |  | 526 | 0.613 | 0.0698 | No |
| 8 | PAPSS2 |  |  | 529 | 0.612 | 0.0935 | No |
| 9 | SSR3 |  |  | 557 | 0.598 | 0.1104 | No |
| 10 | PTGR1 |  |  | 641 | 0.558 | 0.1119 | No |
| 11 | NQO1 |  |  | 732 | 0.522 | 0.1102 | No |
| 12 | CYB5A |  |  | 737 | 0.520 | 0.1297 | No |
| 13 | SLC1A5 |  |  | 757 | 0.514 | 0.1453 | No |
| 14 | PTGES3 |  |  | 763 | 0.512 | 0.1642 | No |
| 15 | EPHA2 |  |  | 791 | 0.502 | 0.1773 | No |
| 16 | GART |  |  | 820 | 0.491 | 0.1898 | No |
| 17 | AOX1 |  |  | 896 | 0.472 | 0.1899 | No |
| 18 | ETS2 |  |  | 923 | 0.466 | 0.2018 | No |
| 19 | SHMT2 |  |  | 1361 | 0.366 | 0.1084 | No |
| 20 | ACP1 |  |  | 1396 | 0.358 | 0.1141 | No |
| 21 | ASL |  |  | 1437 | 0.350 | 0.1180 | No |
| 22 | AHCY |  |  | 1471 | 0.344 | 0.1234 | No |
| 23 | AP4B1 |  |  | 1590 | 0.326 | 0.1072 | No |
| 24 | UGDH |  |  | 1797 | 0.299 | 0.0681 | No |
| 25 | PMM1 |  |  | 1976 | 0.276 | 0.0350 | No |
| 26 | BCAR1 |  |  | 2057 | 0.266 | 0.0258 | No |
| 27 | SLC12A4 |  |  | 2107 | 0.262 | 0.0240 | No |
| 28 | ARG2 |  |  | 2149 | 0.257 | 0.0240 | No |
| 29 | POR |  |  | 2321 | -0.261 | -0.0079 | No |
| 30 | CAT |  |  | 2344 | -0.264 | -0.0029 | No |
| 31 | BPHL |  |  | 2371 | -0.267 | 0.0012 | No |
| 32 | PROS1 |  |  | 2593 | -0.300 | -0.0415 | No |
| 33 | PINK1 |  |  | 2608 | -0.302 | -0.0330 | No |
| 34 | ECH1 |  |  | 2885 | -0.353 | -0.0873 | No |
| 35 | ACOX1 |  |  | 3121 | -0.404 | -0.1294 | No |
| 36 | TNFRSF1A |  |  | 3146 | -0.413 | -0.1190 | No |
| 37 | CYFIP2 |  |  | 3265 | -0.445 | -0.1306 | No |
| 38 | ID2 |  |  | 3664 | -0.654 | -0.2031 | Yes |
| 39 | GABARAPL1 |  |  | 3667 | -0.657 | -0.1776 | Yes |
| 40 | GAD1 |  |  | 3704 | -0.690 | -0.1593 | Yes |
| 41 | TGFB2 |  |  | 3774 | -0.766 | -0.1462 | Yes |
| 42 | IDH1 |  |  | 3842 | -0.832 | -0.1299 | Yes |
| 43 | APOE |  |  | 3864 | -0.860 | -0.1012 | Yes |
| 44 | DDAH2 |  |  | 3895 | -0.936 | -0.0717 | Yes |
| 45 | FBLN1 |  |  | 3909 | -0.965 | -0.0369 | Yes |
| 46 | NINJ1 |  |  | 3929 | -1.015 | -0.0016 | Yes |
| 47 | HES6 |  |  | 3961 | -1.092 | 0.0338 | Yes |
Table: GSEA details [plain text format]

  

Fig 2: HALLMARK\_XENOBIOTIC\_METABOLISM: Random ES distribution      
 Gene set null distribution of ES for **HALLMARK\_XENOBIOTIC\_METABOLISM**
